# Production of gold/silver doped carbon nanocomposites for effective photothermal therapy of colon cancer

**DOI:** 10.1038/s41598-020-64225-8

**Published:** 2020-05-06

**Authors:** Fang Liu, Xiao-di Wang, Shi-yu Du

**Affiliations:** Department of Gastroenterology, China-Japan Friendship Hospital, Beijing, 100029 China

**Keywords:** Colon cancer, Cancer microenvironment

## Abstract

Surgery followed by adjuvant chemotherapy is a reliable therapy for colon cancer, but is associated with side effects and risks. Recent advancements in nanobioengineering in the form of targeted nanoparticles, cubosomes, liposomes, nanosheets, nanorods, quantum dots have generated substantial advancements in theranostics of colon cancer decreasing the cytotoxic drugs’ side effects. We describe a facile mechanism of preparation of hybrid nanocomposite encompassing Au and Ag. Preparation of hybrid nanocomposite is one step process which may be easily escalated. The nanocomposite was characterized using transmission eleactron microscopy, energy dispersive X-Ray spectroscopy, X-ray photoelectron spectroscopy, Fourier transform infra-red spectroscopy, UV-Vis spectroscopy, photoluminescence and cytotoxic studies. *In-vivo* studies were carried out in Balb/c mice. Photothermal heating experiments in HeLa cells were promising and the characterization studies clearly indicated the formation of hybrid nanocomposite. *In-vivo* experiments confirmed the efficacy of treatment, along with involvement of epigenetic regulation, which may be helpful in translation from research to clinical applications.

## Introduction

Colon/colorectal cancer is the fourth most dangerous form of cancer causing high mortalities around the world^1^. Surgical interventions followed by adjuvant chemotherapeutic sessions remain until today, the only option for treatment of solid tumors. Other treatments including transplant of stem cell, immunotherapy, hormonal therapy, targeted drug through nanoparticles have been suggested. However, these may be accompanied by side effects like chances of recurrence of metastatic tumors, unendurable cytotoxicity, non-restricted annexations in various other tissues and extremely low bioavailability of encased drugs^2,3^. Hence, there is an immediate need for the development of new theranostic options for these metastatic tumors with minimal side effects.

Photothermal therapy has emerged as an alternative option for cancer therapeutics and the reasons that make it such an attractive option are manifold. This therapy is highly efficient, can be achieved in a controlled manner and is minimally invasive^2^. Photothermal therapy involves ablation of the cancerous tissues utilizing laser^4–7^, high intensity ultrasonography, microwave or radiofrequency radiation^8,9^. Optically sensitive mediators are used to endogenously absorb the optical energy and transform it into thermal heat using near-infrared (NIR, 650–1025 nm) radiation. The heat energy which is so generated destroys the cellular membranes and causes protein denaturation causing cancer cell death^2,10,11^. However, the procedure is generally restricted by the precision of imaging, applicator utilized for the therapy and the shape of the applicator whether conical or round as this is directly related to energy deposition^4^. Hence for precise and effective therapy, the mediators must absorb energy in the NIR spectrum and must be absorbed at the specific cancerous tissue. These must also facilitate imaging for increasing therapeutic efficacy^12^. Photo thermal therapy utilizes no drug but metallic or semiconductor materials for cancer cell destruction.

Nanobioengineering offers choice of nanoplatforms for the ablation therapy of the solid tumors. These sub-micron or nanosized particles offer great precision in such applications and are designed such that they accumulate precisely at the site of tumor without invading other normal cells or tissues. This is accomplished by passively targeting the particles to the tumor site by vascular fenestrations^13–15^. One of the most frequently reported is the gold based nanoparticles for this photo ablation therapy^16^. Gold based nanoshells, nanoparticles, nano cages, nano clusters have all being used for photothermal ablation, targeted drug delivery and so on^17^. Gold nanoplatforms have generated significant interest due to their submicron size and absorption coefficients. Furthermore, there are also reports on Gandolium based compounds being integrated with gold nanoparticles to prepare a hybrid nanocomposite for photothermal ablation therapy^18–20^.

These studies formed a background for the current research to prepare a gold/silver doped hybrid carbon nanocomposite utilizing a very facile yet effective agent for photothermal therapy against colon cancer. Our study demonstrates a restrained, simple and bio-compatible formation of hybrid gold/silver doped carbon quantum dot nanocomposite without utilizing any supplementary reducing compounds. The hybrid nanocomposite was prepared by addition of a gold precursor to the silver quantum dots. The gold/silver hybrid carbon quantum dot nanocomposite exhibited excellent stability and absorbance at NIR region. They were also noted for the photo ablation therapy by destroying HeLa cells in *in-vitro* studies. *In-vivo* studies in mice demonstrated their ability to reduce the volume of tumors. Hence, to our knowledge, this is a first of its kind study to prepare gold/silver doped hybrid carbon quantum dot nanocomposite for photothermal therapy in colon cancers.

## Materials and Methods

Unless specifically mentioned, all the chemicals were procured from Sigma Aldrich (China).

### Preparation of silver doped hybrid carbon quantum dot nanocomposite

Silver doped hybrid carbon quantum dot nanocomposite was prepared following the protocol as per Gedda *et al*.^21^ and adding necessary modifications. The methodology was being termed as one-pot microwave pyrolysis method which was also reported by^22^. The facile mechanism of synthesis is as follows; AgCl (0.03 mM), N-acetyl-L-cysteine (0.24 mM), sodium citrate (0.16 mM), and Na_2_S_9._9H_2_O (0.16 mM) were added as final concentration to distilled water (20 mL) under ultrasonication. This mixture was moved to a glass tube which could be microwaved and further warmed for 10 min at 220 °C. Following the reaction, the glass tube was brought to room temperature for cooling. After cooling, the subsequent mixture, using a Millipore syringe filter (0.22 μm) was filtered. Consequently, centrifugation of the mixture was done at 2000 rpm for 20 min to remove unreacted materials. Finally, the supernatant comprising silver doped hybrid carbon quantum dot nanocomposite was freeze-dried overnight and further liquefied in Milli-Q water for synthesis of gold/silver doped hybrid carbon quantum dot nanocomposite. The mixture will hence forth be mentioned as Ag-CQD-NC.

### Preparation of gold/Ag-CQD-NC

Chemical reduction of HAuCl_4_.3H_2_O was induced for the preparation of gold/Ag-CQD-NC henceforth to be termed as Au/Ag-CQD-NC. In a typical process, 5 ml of Ag-CQD-NC was added dropwise to 300 µl of HAuCl_4_.3H_2_O (1 mg/ml) followed by rigorous stirring. It was also subsequently heated for 2 hours at 80 °C. The reddish yellow tinged Au/Ag-CQD-NC was for one last time, centrifuged at 14000 rpm for 15 minutes and dispensed in Milli-Q water for further utilization.

### Visualization of Au/Ag-CQD-NC using Transmission Electron Microscopy (TEM)

Morphological analysis of the Au/Ag-CQD-NC and only Ag-CQD-NC was done using a high resolution TEM, JEM-2010HR microscope. On carbon coated copper grids of 5 mm diameter, drops of Au/Ag-CQD-NC and Ag-CQD-NC were placed with care and additional solution was soaked up carefully with absorbent paper. Samples were stained with 1% phosphotungstate solution and the hybrid nanocomposites were viewed at 10000X magnification.

### Energy-dispersive X-ray spectrometer (EDS)

Elemental composition of the Au/Ag-CQD-NC and only Ag-CQD-NC was done using JEOL JSM Scanning electron microscopy.

### Optical Characterization (UV-VIS spectroscopy and Photoluminescence (PL)

Absorbance spectra of the Au/Ag-CQD-NC and Ag-CQD-NC were recorded using a UV-Vis spectrophotometer (Lasany LI-2800). A HORIBA Fluoro Max-4P spectrophotometer was utilized to determine the photoluminescence (PL) spectra at room temperature. 0.03 mg/ml aqueous solutions of Au/Ag-CQD-NC and Ag-CQD-NC were used. The PL studies were conducted for Au/Ag-CQD-NC for different concentrations which were further to be tested in the cytotoxicity and *in-vivo* studies. PL studies were carried out for 0.1, 0.5, 1, 2, 3 and 4 mg/ml of Au/Ag-CQD-NC.

### Fourier transform infra-red (FTIR) spectroscopy and X-ray photoelectron spectroscopy (XPS)

The FTIR spectra were plotted to confirm the functional groups utilizing a Nicolet IR 200 FT-IR spectrometer (Thermo Fisher Scientific). XPS (Thermo Fisher) measurements were completed by means of monochromatic AlKα radiation (hʋ= 1486.6 eV).

### Cell culture and cytotoxicity assay

Rat C6 glioma (C6 cells) were purchased from ATCC China. They were cultured in Dulbecco’s modified Eagle’s medium (DMEM) with the following supplements; 10% fetal calf serum (FCS), penicillin (100 IU/mL), and streptomycin (100 mg/mL). The supplements were added in humidified air containing 5% CO_2_ at room temperature (37 °C). The *in vitro* cytotoxicity was assessed by 3-(4, 5-dimethylthiazol-2-yl)-2, 5-diphenyltetrazolium bromide (MTT) assay. In 96-well plates, 5 × 10^3^ cells/well were seeded and gestated for 72 hours to ensure cell viability on addition of Au/Ag-CQD-NC and Ag-CQD-NC; 0.1 mL of DMEM (control), Au/Ag-CQD-NC (100 μg/mL and 4000 μg/mL) and Ag-CQD-NC (100 μg/mL and 4000 μg/mL) were added and gestated overnight. Then, 150 μL of MTT solution (5 mg/mL) was added to each well and the plate was gestated at 37 °C for 6 h. After adequate gestation, the absorbance was comprehended by means of a microplate reader at 492 nm^21,23^. The measurements were made for three times under identical condition to ensure reproducibility.

### Cell Culture for photothermal heating experiment

Human cervical carcinoma (HeLa) cells were procured from ATCC China. Dulbecco’s Modified Eagle’s Medium (DMEM) was the medium in which the cells were initially cultured adding 10% fetal bovine serum, 100 μg/mL streptomycin which acted as antibiotic or antimycotic, 1% L-glutamine, 1% non-essential amino acids and 1% sodium pyruvate was added to the media. Under optimum conditions (37 °C, 5% CO_2_ atmosphere), cells were harvested in an incubator. The procedure was adapted from Banerjee *et al*.^24^.

### Photothermal heating experiment

For understanding the photothermal effect of the Au/Ag-CQD-NC and Ag-CQD-NC nanocomposites, the following procedure was adapted from Gedda^21^ with necessary modifications. 1, 2 and 4 mg/ml of Au/Ag-CQD-NC and Ag-CQD-NC were added to 1 mL of aqueous solution. These solutions were irradiated utilizing an NIR laser (808 nm, 2 W/cm^2^) for 10 min. To indicate the increase in temperature of the solution, a digital thermometer was used. The control was water without Au/Ag-CQD-NC which was irradiated under the same conditions with the laser (808 nm, 2 W/cm^2^).

The incubated HeLa cells were transferred to 96 well plates with a concentration of 2 × 10^6^ cells in each well in DMEM for 24 hours. The incubated cells were washed with PBS thrice and each time the medium was renewed. Then to each well, 1, 2, 4 mg/ml Au/Ag-CQD-NC and Ag-CQD-NC were added. This was then irradiated with NIR laser (808 nm, 2 W/cm^2^) for 10 min.

The cell viability was assessed using fluorescence co-staining by calcein AM and propidium iodide. Simultaneous fluorescence staining of live and dead cells is assessed by the Live/Dead Cell Double Staining Kit which is procured from Merck Pharmaceuticals (Hong Kong) Ltd. This procedure was adapted from Wang *et al*.^25,26^.

### Histone H3 acetylation

Total histone H3 acetylation was measured by Histone H3 Acetylation Assay Kit (Abcam, China) which allows measurement of global acetylation of histone H3. The assay was performed exactly as recommended in the supplied protocol. % histone acetylation was calculated using the formula: ((Absorbance of treated sample – Absorbance of blank)/ (Absorbance of untreated sample – Absorbance of blank))*100.

### *In-vivo* studies

The *in vivo* experimental protocols were approved by Institutional Animal Care and Use Committee of the China-Japan Friendship Hospital (protocol # 19/345 A). All methods were carried out in accordance with relevant guidelines and regulations, and guidelines under “Guide for the Care and Use of Laboratory Animals” (Institute of Laboratory Animal Resources, Commission on Life Sciences 2011) were strictly followed. 3–5 months old Balb/c female mice were utilized for the experiments. Four groups were designated for the study. They were caged in four cages having 2 each with ample amount of food and water. They were obtained from Vital River laboratories in Beijing, China. The *in-vivo* study protocol was presented in front of the small animal institutional ethics committee and permitted by them before commencing the studies. The technical incorporation from the committee was included in the studies.

Murine colon cancer cells (CT26) were obtained from ATCC, China. 80% of the CT26 cells were trypsinized followed by a wash with PBS. This was further centrifuged at 200 × g for 5 mins. The supernatant was discarded and the washing step was repeated thrice. 5 × 10^6^ cells in 50 µl of PBS were kept ready for injection in mice subcutaneously. The hair was removed from the lower abdominal region of mice and an incision of made in body wall. The cecum was slowly pulled out, kept moist and the cells were injected in the wall of the cecum. Then the cecum was put back slowly. The abdominal layer and the skin were sutured. The procedure was adapted with modifications from Liao *et al*.^27^.

After the tumor volume reaches an average size of 100 mm^3^, one group was injected with irradiated water (100 ml/kg^−1^), another with only Ag-CQD-NC, one with Au/Ag-CQD-NC, one with Au/Ag-CQD-NC (irradiated with NIR laser, 808 nm, 1 W/cm^2^ for 5 minutes) (Wang *et al*.). The mice were kept under constant observation.

### Statistical Considerations

Data were compared using SPSS software and the two-sided student t-test. *p* <  0.05 was considered statically significant.

## Results

### Preparation of the hybrid nanocomposites

**Figure 1:**
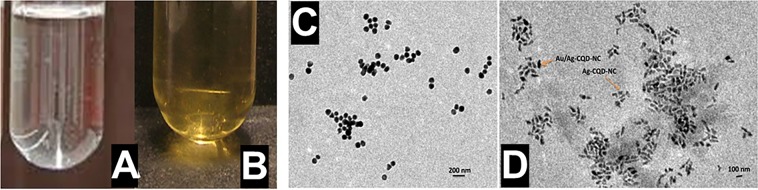
(**A**) Ag-CQD-NC and (**B)** Au/Ag-CQD-NC. Addition of Au metal atoms leads to change in colour. **(C)** Au/Ag-CQD-NC and **(D)** Au/Ag-CQD-NC & Ag-CQD-NC. Au/Ag-CQD-NC is different from Ag-CQD-NC.

The hybrid nanocomposite can be seen in Fig. 1A,B. The Ag-CQD-NC has a yellowish tinge to it which converts into reddish yellow upon interaction with the Au metal ions and consequently the thermal activation may have led to a change in the colour of the solution of the hybrid nanocomposite.

### Visualization of Au/Ag-CQD-NC through TEM

TEM of the prepared Au/Ag-CQD-NC and Ag-CQD-NC exhibits a well-known and interesting trend depicted in Fig. 1C,D. The nanocomposite appears to be spherical but with established smooth edges. Hence, we can say that it’s quasi-spherical in shape. They are homogeneous in distribution and have a uniform separation too. Furthermore, we may hypothesize that as mentioned above the Au^3+^ ions of gold precursor form an electron salt trap for the Ag-CQD-NC leading to initiation of nucleation causing a quenching effect of the Ag-CQD-NC. Therefore, in Fig. 1C,D, we clearly see the high contrast images of Au/Ag-CQD-NC as compared to the low contrast images of Ag-CQD-NC.

### EDS characterization

**Figure 2:**
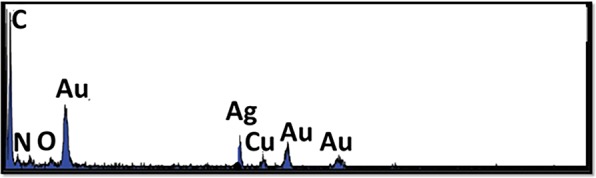
EDS characterization of Au/Ag-CQD-NC.

The EDS spectrum is depicted in Fig. 2. As seen in this figure, the presence of C, O N is seen. Moreover, the presence of Cu, Au and Ag is also visualized. Cu in the sample is probably from the copper grids on which the samples were placed during TEM sample preparation. The images confirm the successful preparation of Au/Ag-CQD-NC.

### UV-Vis spectroscopy and photoluminescence (PL) studies

**Figure 3:**
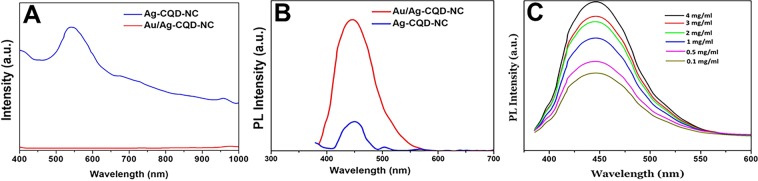
(**A**) UV-Vis spectra of Au/Ag-CQD-NC & Ag-CQD-NC. **(B)** Photoluminescence (PL) spectra of Au/Ag-CQD-NC & Ag-CQD-NC. **(C)** Photoluminescence (PL) spectra of Au/Ag-CQD-NC at different concentrations of nanocomposite.

UV-Vis spectroscopy results are seen in Fig. 3A. Few interesting observations have been made. It was seen that Ag-CQD-NC have an absorption spectra exhibited a strong peak at around 550 nm. After the addition of the Au^3+^ ions from the gold precursor, the peak disappeared due to durable resonance of the plasmon, after which an absorption band is seen in the region of 400–1000 nm. This trend confirms the formation of Au/Ag-CQD-NC from Ag-CQD-NC and as well as its absorption properties in the NIR region of the spectrum.

PL studies exhibit similar interesting trends depicted in Fig. 3B. The PL emission spectra of Au/Ag-CQD-NC is significantly more intense than Ag-CQD-NC. The PL intensity is almost 2.5 times more than Ag-CQD-NC. As verified from the TEM images, this is substantiated by PL studies that on addition of Au metal atoms to the Ag-CQD-NC, the shift in brightness in the PL causes the Au/Ag-CQD-NC to stand out in contrast to the Ag-CQD-NC. The increase in PL intensity may also be a result of localization of electron hole pairs which results from the carbon-oxygen matrix on addition of Au metal atoms^28^. In Ag-CQD-NC the disruption of electron hole pairs occurs due to nucleation at these sites, thereby decreasing the intensity of PL emission spectra caused by quenching.

Furthermore, realising this trend, PL studies were conducted for different concentrations of Au/Ag-CQD-NC to check for PL intensity. The concentration of 0.1, 0.5, 1, 2, 3, 4 ng/ml of Au/Ag-CQD-NC were tested. It was clearly seen from Fig. 3C, that with increase in concentration of Au/Ag-CQD-NC, there is a rise in PL intensity. Hence the three increased doses were used for the other studies. This maximization of intensity on increasing concentration may be due to additional chemical stability provided by Au metal atoms to the hybrid nanocomposite.

### FTIR and XPS studies

**Figure 4:**
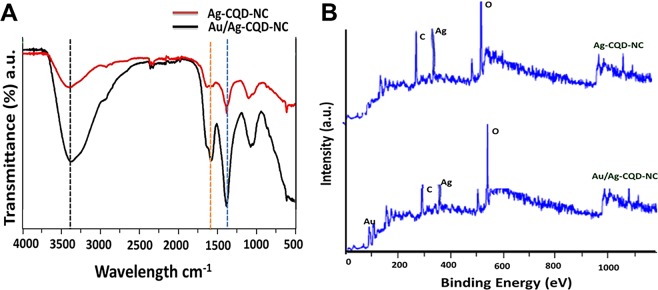
(**A**) FTIR spectra of Au/Ag-CQD-NC & Ag-CQD-NC; **(B)** XPS spectra of Au/Ag-CQD-NC & Ag-CQD-NC.

The FTIR spectrum is seen in Fig. 4A and validates the previous observations. The characteristic N-H stretching mode was found to be present at 3448 cm^−1^. There is a stronger N-H stretching in Au/Ag-CQD-NC than Ag-CQD-NC. The C=O at 1650 cm^−1^ respectively was visible in both groups. The C-OH bond at 1400 cm^−1^ was seen in Ag-CQD-NC and Au/Ag-CQD-NC. It was clear cut indication of formation of nanocomposites.

XPS studies indicated in Fig. 4B confirms the presence of Au, Ag, C, O in the nanocomposite. The binding energy of 88.4 and 86 eV indicates presence of Au atoms^29^. The presence of Ag is indicated by the binding energy of 368 and 374.5 eV^30^. This is an elucidation that both Au and Ag are associated with the hybrid nanocomposite. 284.5, 284.6, 285.0, and 288.4 eV are the peaks exhibited by C, conforming sp^2^ carbon (C=C), sp3 carbon (C–C), C=O, and C–O. The additional 285.0 eV and 288.4 eV peaks endorse the occurrence of functional groups having oxygen^29,31,32^.

### *In-vitro* cytotoxicity

**Figure 5:**
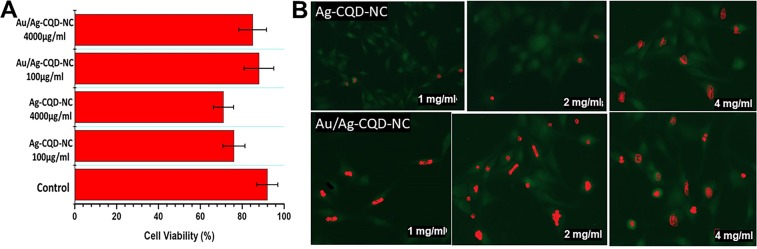
(**A**) Cytotoxicity of Au/Ag-CQD-NC at different concentrations (100 & 4000 µg/ml) & Ag-CQD-NC (100 & 4000 µg/ml). **(B)** Photothermal heating experiments on exposure to 1, 2 & 4 mg/ml of Ag-CQD-NC & Au/Ag-CQD-NC respectively.

The cytotoxicity was tested on rat glioma C6 cells and it was observed that cells were viable even after treatment with 4000 µg/mL of both Au/Ag-CQD-NC and Ag-CQD-NC which according to some studies was quite high dose of nanocomposite treatment. Typically, the cytotoxicity of a nanocomposite depended upon the aberrations it causes in the cells at high dose. It was seen in our studies that cell viability upon exposure to control is 92% which is 76% in case of treatment with Ag-CQD-NC (100 µg/mL) and 71% in case of Ag-CQD-NC (4000 µg/mL). It was also seen that the cell viability was 88% upon treatment with Au/Ag-CQD-NC (100 µg/mL) and 85% upon treatment with Au/Ag-CQD-NC (4000 µg/mL). This is depicted in Fig. 5A. Therefore, it may be safely said that Au/Ag-CQD-NC and Ag-CQD-NC are not cytotoxic.

### Photothermal heating

The photothermal heating experiment revealed that upon irradiation by NIR laser, HeLa cells were exposed to photocatalytic process which severely hampered the cell viability. The photocatalytic process is heat induced targeting of the HeLa cells. The HeLa cells are thermosensitive cells which upon exposure to severe increase in temperature, led to cell death. The hybrid nanocomposite Au/Ag-CQD-NC and Ag-CQD-NC upon induction caused by NIR laser (808 nm, 2 W/cm^2^) led to cell death. It may be appropriate to remind here that almost the same dosage of hybrid nanocomposite when checked for their cytotoxicity in rat glioma c6 cells didn’t exhibit cytotoxicity. Here, when the cells were exposed to 1, 2 and 4 mg/ml of Ag-CQD-NC and Au/Ag-CQD-NC and then irradiated with NIR laser caused cell death indiscriminately as seen in Fig. 5B. The Au/Ag-CQD is an efficient NIR absorber which conveniently upon exposure to NIR laser converts the optical energy into thermal energy. The photothermal property of the Au/Ag-CQD-NC was exhibited when upon irradiation with NIR laser, the temperature of the solutions increased by 19 °C, 20 °C and 23 °C respectively for 1, 2 and 4 mg/ml nanocomposite. The temperature increase of 20 °C and above is sufficient for effective killing of tumor cells^21^. For Ag-CQD-NC, the increase in temperature was 13, 15 and 16 °C respectively for 1, 2 and 4 mg/ml of nanocomposite. The results sufficiently prove that Au/Ag-CQD-NC not only has the ability to quickly transform the NIR laser optical energy to thermal heat and is also an efficient mediator in the conversion and is quite stable too.

The images of the cells stained with calcein AM and propidium iodide exhibit a similar trend. Propidium iodide penetrates the disintegrated cell membrane of the dead cells upon exposure to thermal heat and therefore stains them red. The thermal heat generated by converting the optical energy from NIR laser was effectively transferred to the environment of the cells. Subsequently, local hyperthermia was formed which killed the cancerous cells. Majority of cells are stained red in higher doses of Au/Ag-CQD-NC. Calcein AM stained the live cells green as seen in Fig. 5B. These results demonstrate that Au/Ag-CQD-NC has tremendous potential for use as photothermal agents for effective killing of cancer cells.

### *In-vivo* studies

**Figure 6:**
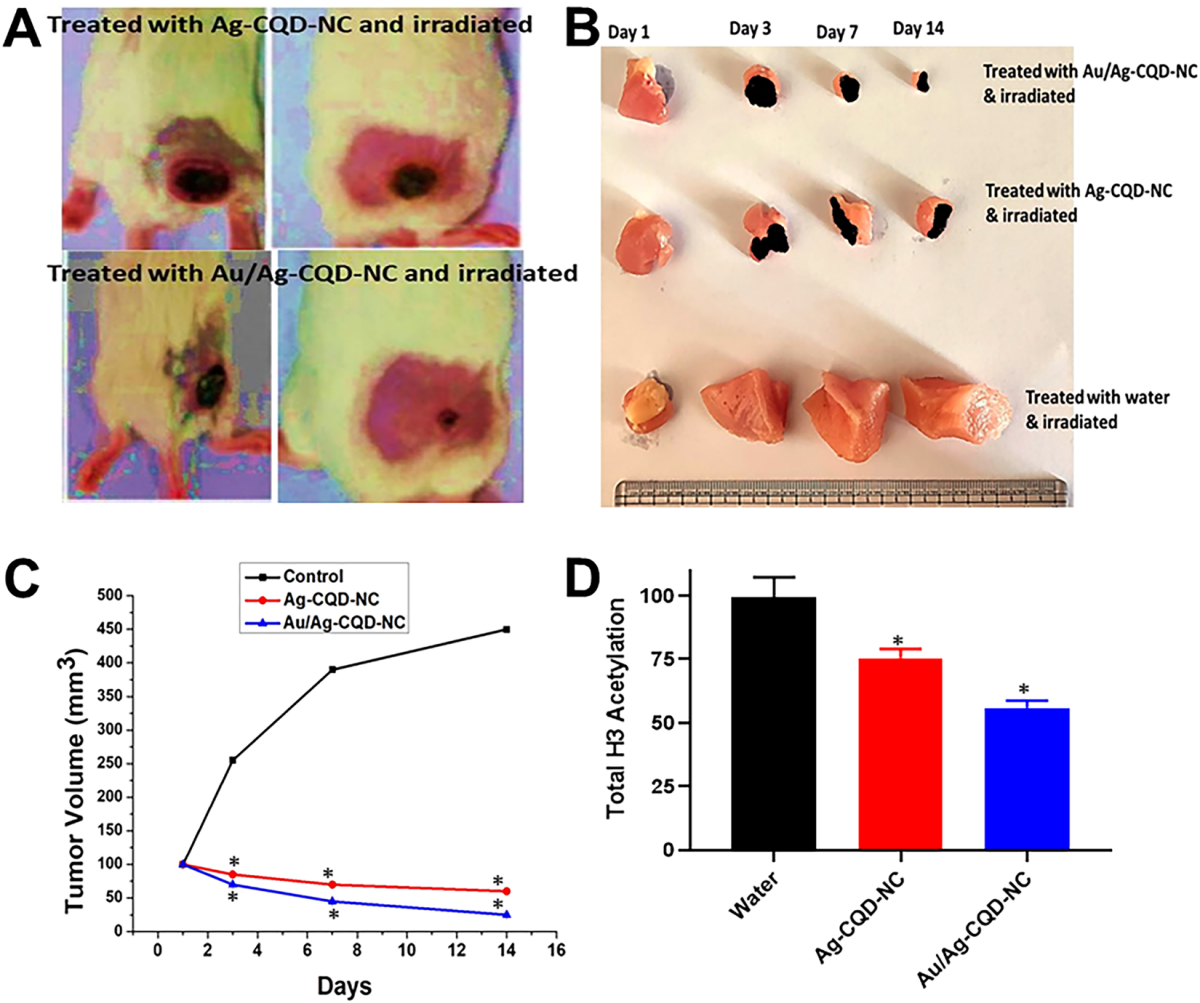
(**A**) Tumors turns black after treatment and irradiation of Au/Ag-CQD-NC and Ag-CQD-NC with NIR laser. **(B)** Sizes of the tumor after treatment and irradiation of Au/Ag-CQD-NC and Ag-CQD-NC with NIR laser. **(C)** Tumor volume after 14 days after the treatment and irradiation of Au/Ag-CQD-NC and Ag-CQD-NC with NIR laser. **(D)** Estimation of total histone H3 acetylation in tumors from different groups. *p < 0.05, compared to control.

Enthused by the *in-vitro* performance of Au/Ag-CQD-NC, we proceeded for the *in-vivo* implantation of the Au/Ag-CQD-NC and Ag-CQD-NC in the Balb/c mice. We saw an expected but a quite interesting trend. Upon irradiation with NIR laser the mice which were injected with Au/Ag-CQD-NC, the tumor ablated and turned black as seen in Fig. 6A. The tumor increased in the mice injected with water and tumor size was reduced in ones treated with only Ag-CQD-NC but was much less than Au/Ag-CQD-NC as seen in Fig. 6B. The tumor turned black in this case too. This was a direct evidence of elevation of temperature upon irradiation with NIR laser. After a few days, the blackened area of tumor in mice treated with Au/Ag-CQD-NC and Ag-CQD-NC healed. But, an important point to be noted here is in our study, the body temperature of the mice was controlled constantly between 42 °C- 45 °C using a temperature cooling pad during the irradiation. The temperature was never allowed to go too high to avoid fatality in mice.

The tumor volumes were also calculated as depicted in Fig. 6C. The day of irradiation with laser was denoted as day 1. After intervals of 3, 7 and 14 days the tumor volumes were measured. In the mice injected with water the tumor volume after a time period of 14 days, increased to 450 mm^3^, the starting volume being 100 mm^3^. In the mice treated with Ag-CQD-NC, the tumor volume decreased to 60 mm^3^, the starting volume being 100 mm^3^. In the mice treated with Au/Ag-CQD-NC, the tumor decreased to a mere 25 mm^3^. This is a clear cut indication that upon irradiation with NIR laser both the hybrid nanocomposites caused regression of tumor volume. The body weight of the mice decreased compared to the control group which after a few more days increased gradually and became comparable with control group. This may possibly due to the ablation of the tumor which caused shrinking in the size. There were no evidences of any toxicity or side effects in the mice compared with the control group. Additionally, there were no deaths reported in the mice too during the experiment or the treatment. Finally, we evaluated the H3 histone acetylation given the recent interest in epigenetic regulation of tumor progression and found that the H3 histone acetylation was markedly reduced by Ag-CQD-NC and even more so by Au/Ag-CQD-NC. Thus the action of nanocomposites involves epigenetic regulation.

## Discussion

It has been studied that carbon quantum dots or the CQDs have surface which are filled with functional groups that may function as electron donor or acceptors^33^. Such architecture on the surface may qualify as nucleation site for the attachment of metallic nanoparticles. In the current study, the presence of carboxyl, hydroxyl and amine moieties on the surface of the CQDs may allow for attachment of Au and Ag and their growth and finally leading to the formation of Au/Ag-CQD-NC, a hybrid nanocomposite. Moreover, these functional groups may lead to reduction of metals salts of Au and Ag, leading to the nanocomposite formation^34^. They also double up as stabilizers protecting the hybrid nanocomposite from accumulation and oxidation.

We hypothesize that in our study, Ag-CQDs acts as electron donor that reduces Au^3+^ to Au metal atoms to form metal nanocomposite. This was activated thermally which led to the growth and formation of Au/Ag-CQD-NC. We further hypothesize that perhaps the Au3+ ions electrostatically interacted with the carboxyl, hydroxyl and amine moieties of Ag-CQD-NC to form the foundation of Au/Ag-CQD-NC. The change of the colour of Ag-CQD-NC from silver-grey to reddish-yellow confirms the hypothesis. Moreover, the distinct difference in contrast in the images as visualized by TEM reconfirms it. The introduction of Au metal atoms is also confirmed by the FTIR, PL, UV-Vis spectroscopy and XPS studies. The physical characterization

The photothermal therapeutic treatment is evident as the local hyperthermia generated at the site of cancer effectively killed the HeLa cells. Solutions of calcein-AM and propidium iodide (PI) are incorporated in the live-dead assay kit that pigments live and dead cells, respectively. Calcein-AM is the acetoxymethyl ester of calcein which is lipophilic and therefore highly permeable through the cell membrane. Nevertheless, this dye by itself is not a fluorescent molecule; the live cell generates calcein from Calcein-AM by the enzyme esterase which emits green fluorescence strongly. Consequently, calcein-AM particularly stains live cells. On the other hand, propidium iodide is a nucleus staining dye that cannot penetrate a live cell membrane. It penetrates the dismantled areas of dead cell membrane and reaches the nucleus to intercalate with DNA double helix emitting red fluorescence. Both these fluorescent dyes may be excited with 490 nm light, hence concurrent observation of live and dead cells was possible with a fluorescence microscope. The death of maximum no. of cells on exposure with NIR irradiated Au/Ag-CQD-NC was indicated by the propidium iodide stained cells.

Cytocompatibility or biocompatibility plays a significant part in deciding the applicability of nanocomposites as therapeutic agents. The hybrid nanocomposite should not be cytotoxic. Although there are huge no of citations reporting *in-vivo* distribution of nanocomposite and *in-vitro* cytotoxicity of the same, the fundamental mechanism of cytotoxicity has still not been clearly understood. It has been seen that usually the major side effect of any kind of nanobioengineering products, be it nanoshells, nanorods, nanodots, CQDs, liposomes, cubosomes and an whole assay of other is unendurable cytotoxicity. Hence, there is a persistent need for reduction of cytotoxicity. It was correctly inferred from the studies that Au/Ag-CQD-NC was lesser cytotoxic than Ag-CQD-NC. One of the major reasons of this non-cytotoxic behaviour is that Au nanoparticles are routinely used for drug delivery in human as well as murine cells and are known to be ideally suited for the purpose^35^. The cytotoxicity induced by the silver nanoparticles may be shielded by the introduction of Au^3+^ ions which changes to Au metal atoms and forms the nanocomposite. This may be explained by the fact although gold and silver are inert biomaterials when in bulk size^35^ but when they are in the submicron form; they may bind to biological molecules. This may result in generation of side effects in cells thereby resulting in cell toxicity. The cell viability assay portrayed an interesting trend of cytotoxicity of the different concentrations of Au/Ag-CQD-NC and Ag-CQD-NC.

Effective targeting at the site of tumor in the *in-vivo* studies is exhibited by the blackening and reduction the sizes of tumor of mice treated with Au/Ag-CQD-NC and Ag-CQD-NC. The greater reduction in size of tumor in case of Au/Ag-CQD-NC (58%) when compared with Ag-CQD-NC may probably be due to the synergistic effects of gold and silver nanoparticles acting together in a hybrid nanocomposite. The blackening of the tumor may additionally refer to a significant increase in local temperature which may indicate towards mortality of the mice but an additional effort towards maintaining body temperature may result in avoiding it, as was in this study. Furthermore, we provide epigenetic regulation and this is a novel and emerging concept in the field of nanotechnology with not enough attention^36^.

Summarizing, we have prepared Au/Ag-CQD-NC successfully using an extremely facile mechanism which have been used for photothermal therapy of cancer both *in-vivo* and *in-vitro*. The hybrid nanocomposite was prepared by reduction of Au^3+^ ions from the gold precursor to Au metal atoms which have the ability to successfully create local hyperthermia^37,38^ and destruction of tumor cells. But the fact remains that research on these photothermal therapy hybrid nanocomposites in still in nascent stage in respect to translation in clinical research. Preclinical studies have to be undertaken to assess further selectivity and well as efficient targeting. Although the preliminary research in our study has indicated that there is extremely low cytotoxicity, this is a dark area that may effectively bring down the possibility of a clinical translation. However, it may be added that if proven otherwise, this may bring down the injected dosage for therapeutic purposes. However, the existing comprehensive data exhibit a clear cut advantage of the hybrid nanocomposite over ongoing therapies. Large animal studies have to be undertaken to ease the shift from research table to clinics.

## Data Availability

All the data generated during this study is included within this manuscript.
